# RTK and PPK GNSS Positioning Accuracy Assessment for UAVs Using a PPS-Synchronized Robotic Total Station

**DOI:** 10.3390/s26154907

**Published:** 2026-08-03

**Authors:** Alessandra Beni, Luca Bigazzi, Lapo Miccinesi, Andrea Cioncolini, Andrea Bulletti, Lorenzo Pagnini, Enrico Boni, Massimiliano Pieraccini

**Affiliations:** Department of Information Engineering (DINFO), Università degli Studi di Firenze, 50121 Firenze, Italy; alessandra.beni@unifi.it (A.B.); luca.bigazzi@unifi.it (L.B.); lapo.miccinesi@unifi.it (L.M.); andrea.cioncolini@unifi.it (A.C.); andrea.bulletti@unifi.it (A.B.); lorenzo.pagnini@unifi.it (L.P.); enrico.boni@unifi.it (E.B.)

**Keywords:** drone navigation, GNSS, PPK, PPS synchronization, RTK, sensor synchronization, total station, trajectory validation, UAV positioning

## Abstract

**Highlights:**

**What are the main findings?**

- A PPS-synchronized Total Station methodology is proposed to directly assess RTK and PPK drone positioning accuracy under dynamic flight conditions.
- Under optimal GNSS conditions, the tested u-blox RTK and RTKLIB-EX PPK solutions provide comparable accuracy; after GNSS degradation, the PPK solution remains in float or degraded states for longer and exhibits larger transient errors.

**What are the implications of the main findings?**

- The choice between RTK and PPK for drone applications should consider not only nominal positioning accuracy, but also reconvergence behavior during dynamic operations.
- The proposed VESTA-based synchronization architecture enables accurate comparison between GNSS positioning solutions and independent reference measurements and can be extended to additional scientific payloads.

**Abstract:**

Accurate drone positioning is essential for applications such as photogrammetric surveying and environmental monitoring. Among differential GNSS techniques, Real-Time Kinematic (RTK) and Post-Processed Kinematic (PPK) are widely adopted to achieve centimeter-level positioning accuracy. However, their performance is typically assessed indirectly through the quality of derived products rather than by comparison with independent ground-truth measurements under dynamic flight conditions. This study presents a direct comparison of RTK and PPK positioning for drone applications using a robotic Total Station (TS) as an independent reference. The experimental platform is based on the Versatile External Synchronization and Telemetry Architecture (VESTA), which integrates differential GNSS receivers, an inertial measurement unit, and Coordinated Universal Time (UTC)-synchronized data logging. A synchronization procedure based on the GNSS Pulse-Per-Second (PPS) signal and the controlled motion of a TS prism was developed to accurately align GNSS and TS measurements. Flight experiments show that RTK and PPK achieve comparable accuracy when the reported positioning quality is optimal. However, for the tested short-baseline datasets and the RTKLIB-EX configuration, the PPK solution remained in float or degraded states for longer following GNSS degradation, resulting in larger transient errors than the real-time u-blox solution. These findings highlight that the choice between RTK and PPK should consider not only nominal positioning accuracy but also reconvergence behavior under dynamic operating conditions.

## 1. Introduction

Precise positioning and georeferencing are essential in a wide range of applications, including environmental surveying, photogrammetry [1,2], and structural health monitoring [3,4]. This requirement is particularly critical in unmanned aerial vehicle (UAV) applications, where accurate trajectory estimation remains challenging due to the highly dynamic nature of flight operations. Reliable position information is fundamental when integrating scientific payloads such as radar [5,6,7,8] or LiDAR [9,10,11], whose measurements strongly depend on the accurate knowledge of the sensor trajectory for correct environmental sensing and data interpretation.

When high-accuracy positioning is required, Differential Global Navigation Satellite System (GNSS) techniques are commonly adopted. These approaches rely on two receivers: a base station located at a known position and a rover whose coordinates are estimated relative to the base. Differential GNSS solutions can be broadly classified according to the processing strategy adopted, namely Real-Time Kinematic (RTK) and Post-Processed Kinematic (PPK).

In RTK mode, the base station continuously transmits correction data to the rover through a communication link, enabling real-time estimation of the rover position with centimeter-level accuracy [12]. Conversely, in PPK mode, the two receivers operate independently during data acquisition, and the differential corrections are applied during a post-processing stage, eliminating the need for a communication infrastructure during the survey, while potentially improving robustness in challenging operating conditions.

Several studies have investigated the adoption of RTK and PPK techniques for drone tracking in photogrammetric surveying, where accurate vehicle positioning is crucial for obtaining reliable georeferenced products and high-quality 3D reconstructions. Conventional photogrammetric workflows typically rely on indirect georeferencing through Ground Control Points (GCPs), which must be deployed and surveyed prior to data acquisition. Although this approach can achieve accuracies better than 5 cm under favorable conditions, it increases operational costs and survey duration and may be impractical in inaccessible or environmentally sensitive areas. For these reasons, RTK and PPK have been extensively evaluated as alternatives or complements to GCP-based workflows, often demonstrating comparable or superior georeferencing performance [1,13,14], particularly in forested or difficult-to-access environments where GCP deployment is not feasible.

Most existing studies, however, assess positioning performance indirectly through the quality of derived photogrammetric products, such as ortho-mosaics, digital elevation models, or three-dimensional reconstructions [2,15]. Consequently, the actual positioning error of the UAV is rarely quantified through direct comparison with an independent reference trajectory, especially under dynamic flight conditions.

Beyond photogrammetry, RTK and PPK techniques have also been investigated in other applications requiring precise positioning. Jemai et al. [16] evaluated both approaches for offshore wind turbine installation, while Nguyen and Vu [17] compared RTK and PPK solutions for structural monitoring applications. Using a mechanical reference system under static conditions, they reported comparable positioning performance for the two techniques. Nevertheless, experimental studies providing a direct and rigorous validation of RTK and PPK positioning accuracy for dynamic UAV trajectories remain limited.

Furthermore, an often-overlooked source of uncertainty in such comparisons is the temporal synchronization between the positioning system under test and the reference measurement system. Even when both systems individually provide high-accuracy coordinates, residual timing offsets can introduce significant discrepancies during dynamic manoeuvres, leading to an overestimation of the positioning error.

Compared with [18], in which VESTA was introduced as a dual-RTK and IMU-based system for PPS-synchronized payload pose estimation, the present work introduces two main extensions. First, VESTA is configured to record raw GNSS observations, enabling the computation of PPK solutions in addition to real-time RTK positioning. Second, a PPS-synchronized robotic Total Station (TS) is integrated as an independent reference system through a controlled motion of the reflective prism. This allows RTK and PPK trajectories to be directly compared with an external ground-truth trajectory under dynamic flight conditions.

To address these limitations, this article presents a systematic experimental evaluation of RTK and PPK positioning performance for UAV applications based on direct trajectory measurements. The Versatile External Synchronization and Telemetry Architecture (VESTA) [18] is employed as a telemetry platform integrating an inertial measurement unit (IMU) and dual GNSS-RTK receivers. All measurements are referenced to Coordinated Universal Time (UTC) through GNSS-derived timing information, while raw GNSS observations are logged to enable post-processed kinematic solutions.

A robotic TS is used to track the UAV during flight and provide an independent ground-truth reference trajectory [18,19,20]. Owing to its millimetre-level nominal accuracy, the TS can be considered a reliable reference for the evaluation of positioning errors. Unlike previous studies that infer positioning quality from secondary products, the proposed methodology directly compares RTK- and PPK-derived coordinates against the reference trajectory, enabling a quantitative characterization of positioning performance under realistic dynamic conditions.

A key contribution of this work is the development of a synchronization procedure based on the GNSS Pulse-Per-Second (PPS) signal and a controlled movement of a target mounted on the UAV. This approach ensures precise and automated temporal alignment between GNSS and TS observations, minimizing synchronization-induced errors that would otherwise affect the comparison. Moreover, the PPS-based synchronization framework provides a common time reference that can be readily extended to additional scientific payloads, enabling the integration of independent sensors within a unified timing architecture.

The main original contributions of this work are:

i. The development of a PPS-based synchronization procedure for aligning Total Station and GNSS measurements;
ii. The extension of the VESTA to support raw GNSS logging for PPK processing;
iii. The direct experimental comparison of RTK and PPK drone positioning against a synchronized independent reference trajectory under dynamic flight conditions.

## 2. Materials and Methods

**Figure 1:**
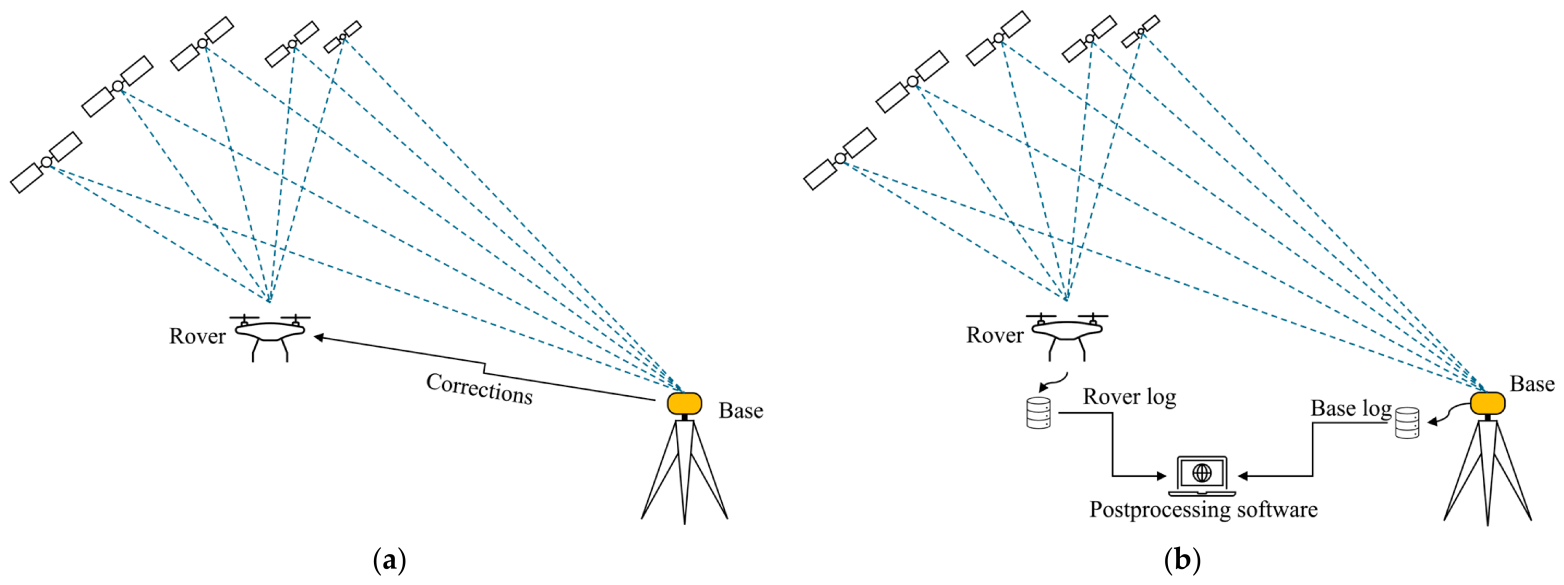
Schematic representation of the GNSS data flow for the two positioning strategies: (**a**) Real-Time Kinematic (RTK), based on real-time correction transmission from the base station to the rover; (**b**) Post-Processed Kinematic (PPK), based on offline processing of raw GNSS observations recorded by both receivers.

RTK and PPK are two GNSS positioning techniques based on differential measurements that differ primarily in their processing strategy. In both approaches, a rover receiver, whose position is to be determined, and a base station simultaneously receive GNSS signals from the same satellites. Figure 1 schematically illustrates the data flow for the RTK (a) and PPK (b) processing modes.

In the RTK approach (Figure 1a), a communication link between the base station and the rover is required. Through this link, typically implemented using a dedicated radio protocol, the base station transmits real-time correction data to the rover, enabling real-time position estimation.

In contrast, the PPK approach (Figure 1b) does not require any communication link during data acquisition. Instead, GNSS observations are independently recorded by both the rover and the base station and processed offline after the survey. The absence of a communication infrastructure can be advantageous, particularly when operating over long distances or in areas with limited connectivity. However, sufficient onboard storage is required to record the raw GNSS observations for the entire acquisition period.

Although RTK requires a more complex system architecture due to the real-time communication link, it provides centimeter-level positioning in real time. PPK, on the other hand, offers greater operational flexibility at the expense of delayed position availability, since the processing is performed after data collection. While both techniques are capable of achieving centimeter-level accuracy under favorable conditions, differences in processing strategy may affect positioning performance during dynamic drone operations. For this reason, a quantitative comparison between RTK and PPK solutions is presented in this work.

### 2.1. Experimental Equipment

The Versatile External Synchronization and Telemetry Architecture (VESTA) (VESTA, Florence Robotics, Florence, Italy) [18] is an electronic board designed to be mounted on multicopter drones as an external telemetry unit. It is capable of independently estimating position and attitude with centimeter-level accuracy.

**Figure 2:**
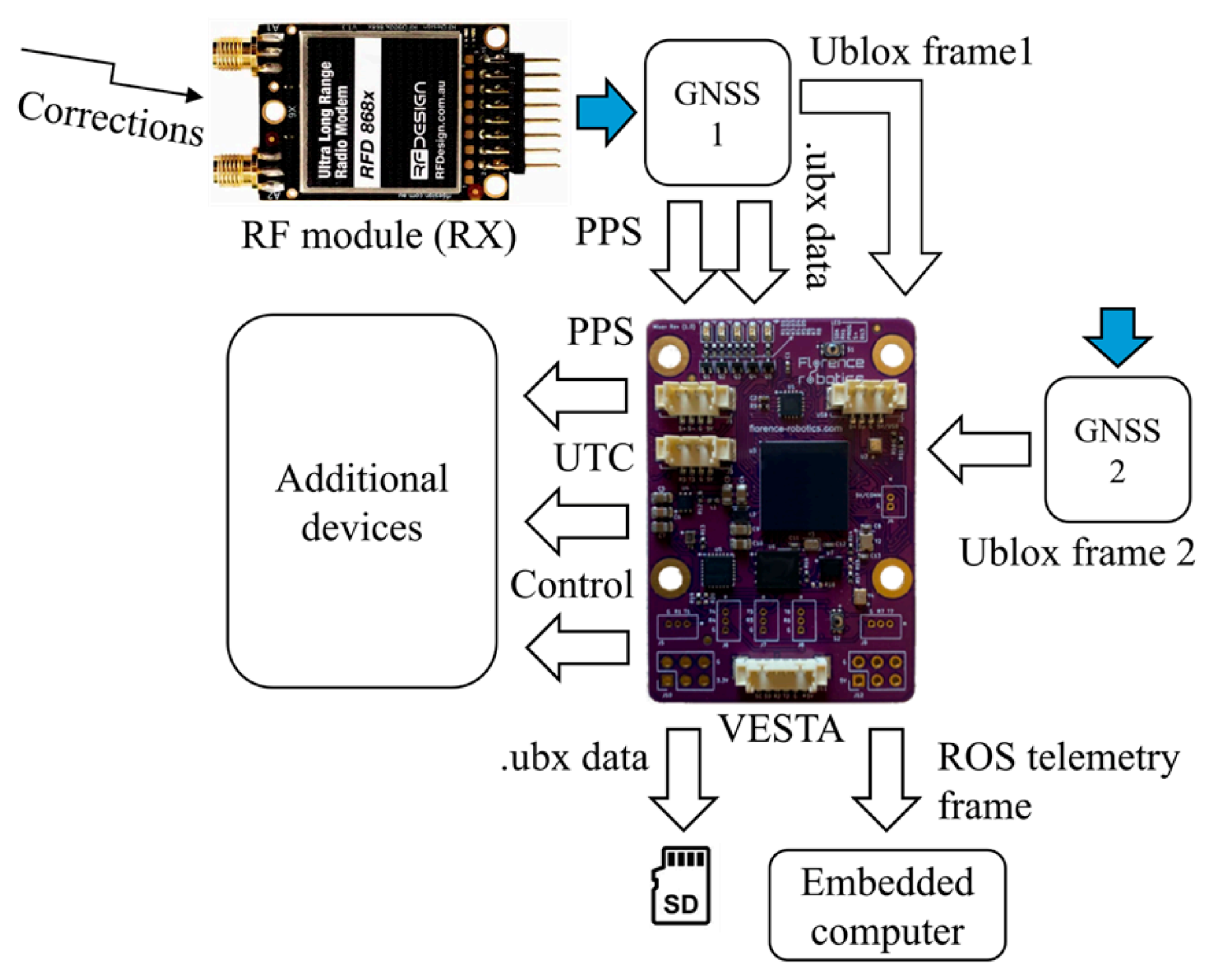
Functional block diagram of VESTA operating with two GNSS receivers.

VESTA is based on a 600 MHz ARM Cortex-M7 microcontroller (i.MX RT1062, NXP Semiconductors N.V., Eindhoven, Netherlands) and is equipped with an ICM-42688P inertial measurement unit (IMU) from TDK InvenSense, San Jose, California. The board can control external devices and acquire signals from several external components or sensors through the microcontroller input/output pins. Figure 2 shows a functional block diagram of VESTA. Two u-blox ZED-F9P GNSS (u-blox AG, Thalwil, Switzerland) receivers are connected to VESTA via UART. Both receivers receive RTK corrections from the radio frequency (RF) module and transmit u-blox messages to VESTA, including GNSS information, such as position and accuracy; RTK information, such as relative position, accuracy, and fix status; and Coordinated Universal Time (UTC). VESTA acquires the Pulse-Per-Second (PPS) signal and the raw GNSS observations from GNSS1. Position and attitude data are transmitted to an onboard computer through a UART channel and saved using the Robot Operating System (ROS) (ROS, Open Source Robotics Foundation, Inc., San Jose, CA, USA). The raw GNSS data, in *ubx* binary format, including the *RXM-RAWX* and *RXM-SFRBX* messages, are saved on a microSD card. These messages contain, respectively, the raw GNSS observables, such as pseudo range, carrier phase, Doppler, and C/N0, and the satellite navigation data required to reconstruct the broadcast ephemerides. The *ubx* logs were converted to receiver-independent exchange (RINEX) format.

The PPS signal, generated by the u-blox GNSS receiver, is a square wave with a duration of 80 ms. Its rising edge is aligned with the beginning of each UTC second with a timing accuracy of approximately 2 ns. For this reason, the PPS signal was selected as the synchronization reference. For example, the IMU timestamp is resynchronized at each PPS pulse [18]. With the same principle, other external sensors can also be synchronized with GNSS time.

**Figure 3:**
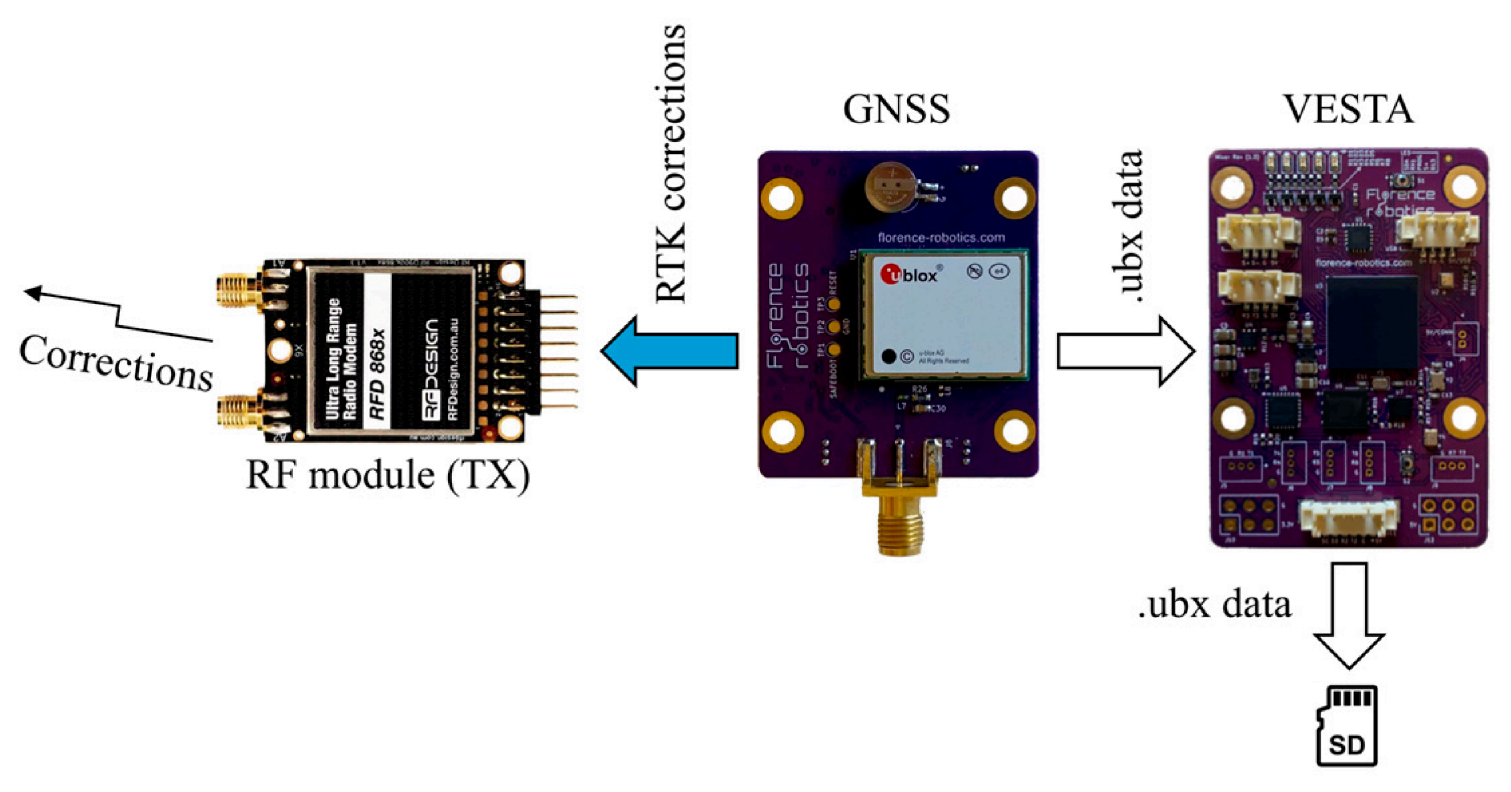
Block diagram of base station used for PPK logging and for RTK correction.

Figure 3 shows the block diagram of the base station used in the experiment. A GNSS receiver was configured to provide both RTK corrections and raw GNSS observations. The RTK corrections were transmitted to the rover through an RF module, whereas the raw GNSS observations were saved on a microSD card connected to VESTA.

The RTK corrections were processed directly by the u-blox receiver, while the PPK dataset, including both base-station and rover observations, was processed using RTKLIB-EX 2.5.0. RTKLIB is an open-source software package for standard and high-precision GNSS positioning [21]. It provides a portable C library together with a set of applications for GNSS post-processing.

The PPK solution was obtained using RTKPOST utility configured in kinematic mode, using the rover and local base station observations as differential inputs. The processing was performed on the L1 + L2 frequencies. An elevation mask of 15° was applied, and the broadcast ephemerides derived from the RINEX navigation files were used. The quality of the solution was assessed using the RTKLIB Q flag and the estimated standard deviations in the North, East, and vertical components.

The reference PPK solution used forward kinematic processing with dynamics enabled; dual-frequency observations from GPS, GLONASS, Galileo, QZSS, and BeiDou; a 15° elevation mask; continuous ambiguity resolution with a validation-ratio threshold of 3.0; and broadcast ephemerides. GLONASS ambiguity resolution was disabled. To assess sensitivity to the processing configuration, the same raw base and rover observations from flights (a) and (b) were reprocessed by changing one element at a time: the elevation mask (10° or 20°), the ambiguity-resolution mode (continuous or fix-and-hold), and the constellation/orbit products. To isolate the ephemeris effect without simultaneously changing the satellite constellation, a controlled GPS-only comparison was performed using broadcast ephemerides and IGS final 15 min orbit and 30 s clock products for 25 May 2026. All other processing settings were kept unchanged.

It is important to note that, in this experimental setup, both the RTK corrections and the raw GNSS observations used for PPK were generated by the same base-station GNSS module, which was located relatively close to the drone. However, the PPK solution could also be computed using observations from a third-party base station. Conversely, using RTK corrections from a different base station would require a suitable real-time communication infrastructure to transmit the correction stream to the rover.

**Figure 4:**
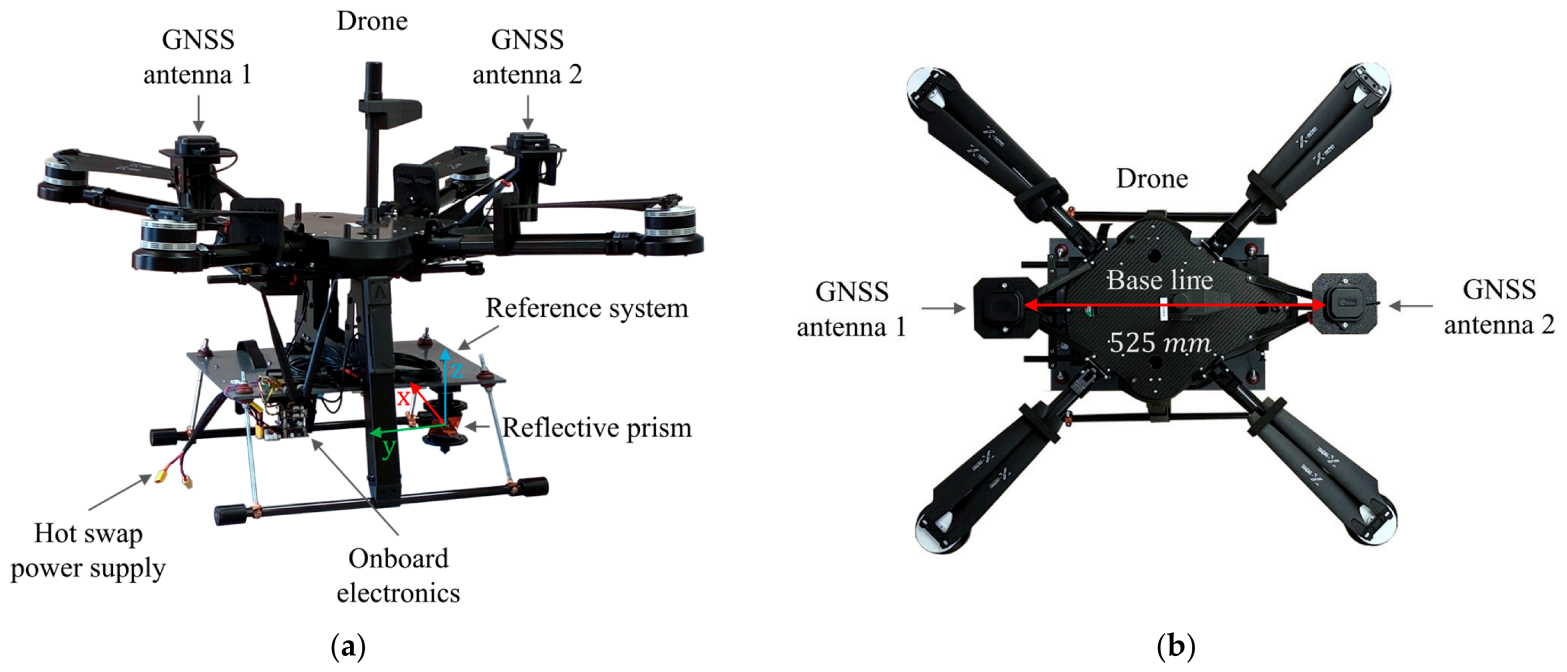
Picture of the drone equipped with the VESTA system: (**a**) lateral view with the detail of GNSS antennas, onboard electronics and the RP; (**b**) upper view with the detail of the horizontal baseline of the GNSS.

Figure 4 shows the experimental drone platform equipped with the VESTA system and a reflective prism (RP) used as the TS target. The two GNSS antennas were mounted on the upper part of the drone using two 3D-printed supports, resulting in a horizontal baseline of 525 mm, as shown in Figure 4b. The RP was installed below the drone. The onboard electronics were powered by an external battery through a power management board designed to enable hot-swapping during operation.

### 2.2. Total Station and Synchronization Principle

A TS is an electronic-optical instrument capable of performing high-precision distance measurements, typically with millimetre-level accuracy. The TS emits a laser signal and determines the distance to a target based on the signal time-of-flight. In this study, a Leica Nova MS60 (Leica Geosystems AG, Heerbrugg, Switzerland) [20,22] was used. This instrument is capable of continuously and automatically tracking the RP (see Figure 4a) and providing its position at a frequency of 5 Hz. The TS measurements are used as reference data to evaluate the performance of RTK and PPK positioning solutions.

To enable a meaningful comparison between positioning datasets, all measurements must be associated with synchronized timestamps, i.e., expressed within the same time reference system. The TS assigns timestamps using its internal clock, which operates as a millisecond-resolution counter. However, this counter is reset whenever the instrument is powered off and restarted from zero after rebooting, making direct synchronization with GNSS-derived timestamps unreliable. To overcome this limitation, an automatic synchronization procedure was developed based on the GNSS Pulse-Per-Second (PPS) signal and the controlled mechanical movement of an RP.

**Figure 5:**
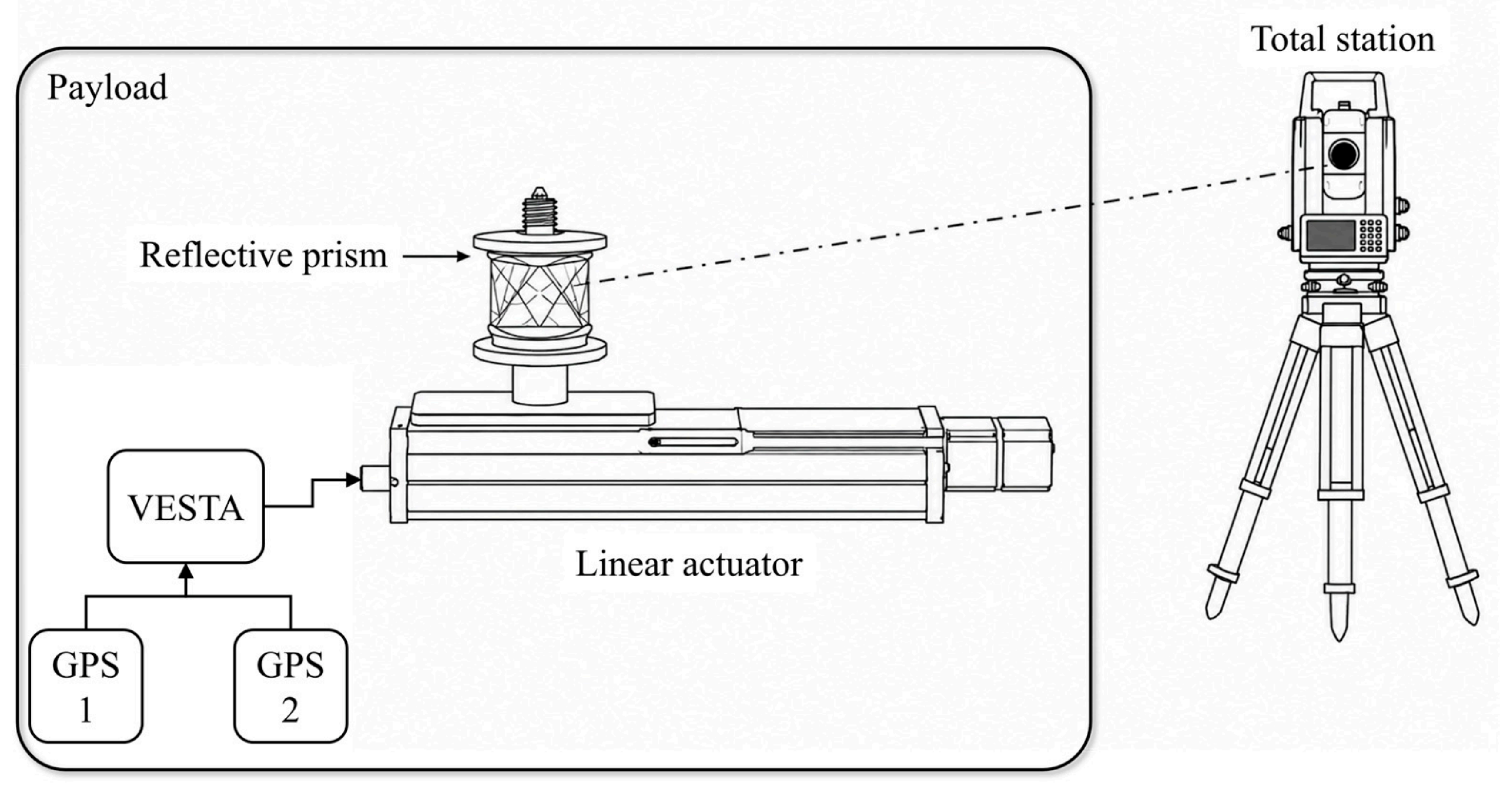
Scheme of the equipment used for automatic synchronization using the PPS signal and TS measurement of RP mechanical movement.

A linear actuator of approximately 8 cm length was mounted on the drone, as shown in Figure 5. The actuator supports the RP that can be tracked by the TS. The synchronization procedure operates as follows. The linear actuator is driven by a periodic square-wave signal. Each rising edge of the signal triggers one actuator step; thus, the signal frequency determines the actuator speed. The square wave is generated by VESTA using the timer-based “tone()” function provided by the Arduino framework [23]. In addition, VESTA records the time delay between the most recent PPS event and the start of the square-wave generation, allowing the actuator motion to be related to the GNSS time reference.

**Figure 6:**
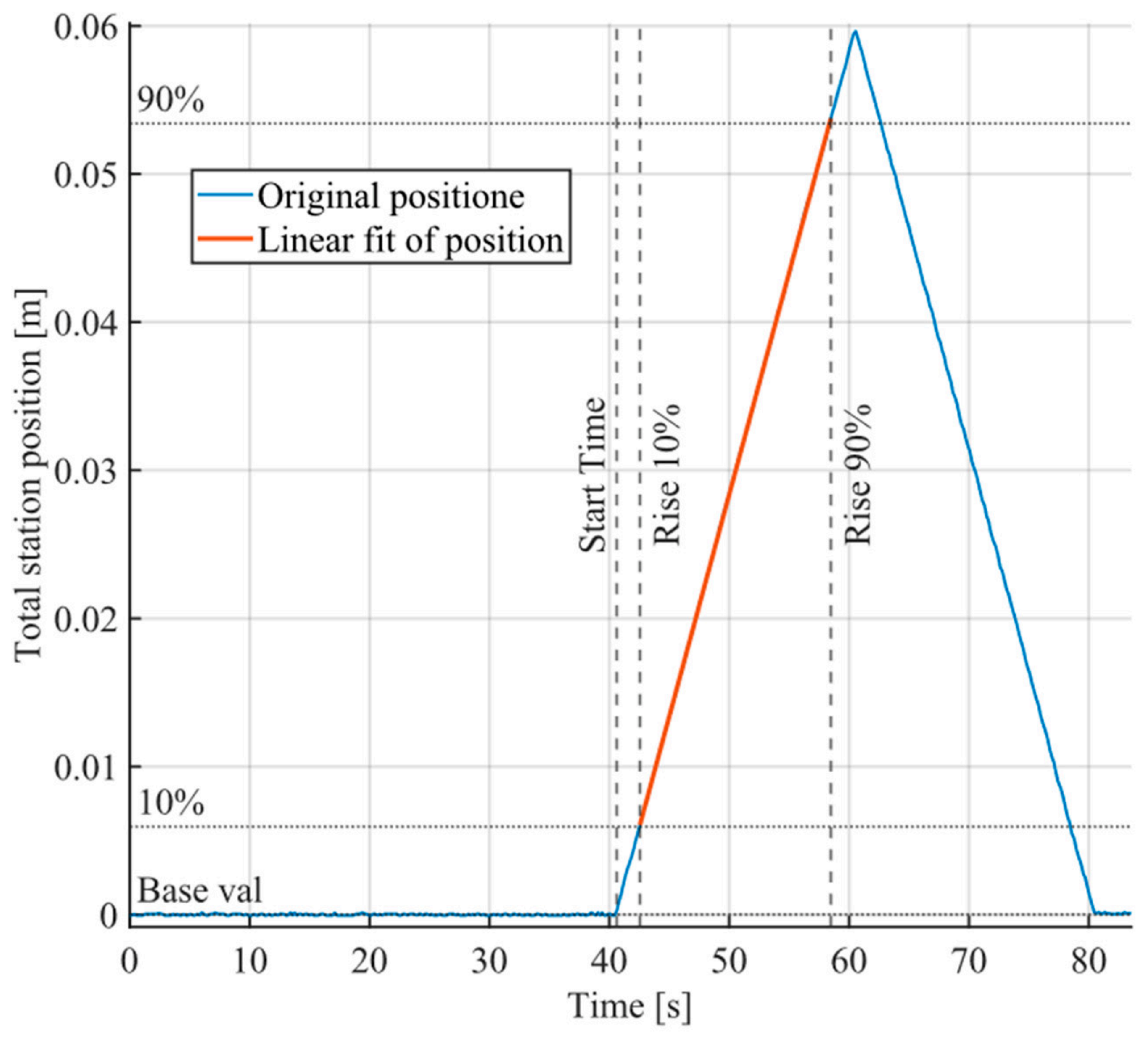
Example of the TS measurements used for the automatic synchronization procedure. The RP displacement produced by the linear actuator is identified from the recorded trajectory, and the ramp start time is estimated by fitting the displacement ramp. This time instant is then associated with the PPS event in the TS time reference.

The TS continuously records the prism position using its own internal time reference. An example of the recorded prism displacement is shown in Figure 6. Since the start of the actuator movement is related to the PPS signal, the corresponding TS timestamp can be identified from the displacement profile and associated with the PPS event.

Because the TS acquisition frequency is limited to 5 Hz and the measurements are affected by noise, the movement start time cannot be reliably identified manually. Therefore, an automated statistical procedure was developed. The TS acquisition begins while the prism is stationary. The mean position during this static interval is computed and used as the baseline value, denoted by ($p_{0}$). The beginning of the displacement ramp is identified as the first sample exceeding a predefined threshold. This threshold is defined as (3σ), where (σ) is the standard deviation of the position measurements during the static interval.

The end of the ramp is determined by identifying the maximum displacement reached by the actuator. Since the actuator moves at an approximately constant velocity, the prism position measurements $p={\left(p_{1},p_{2},\ldots \right)}^{T}$ can be modeled by a linear relationship:

(1) p=m⋅t+q,

where $t={\left(t_{1},t_{2},\ldots \right)}^{T}$ is the corresponding time vector, and m and q are the slope and intercept of the displacement ramp, respectively.

A linear regression was applied to the ramp measurements, and the parameter estimates were obtained as

(2) $$\left(\begin{array}{c} \hat{m}\\ \hat{q} \end{array}\right)={\left(T^{T}T\right)}^{-1}T^{T}\cdot p,$$

with

(3) $$T=\left(\begin{array}{cc} t_{1} & 1\\ t_{2} & 1\\ \ldots & \ldots \end{array}\right).$$

To minimize the influence of acceleration and deceleration phases at the beginning and end of the actuator movement, the regression was performed using only the samples corresponding to 10–90% of the ramp length.

Once the parameters $\hat{m}$ and $\hat{q}$ have been estimated, the ramp start time $t_{start}$ is determined as the instant at which the fitted line intersects the baseline position $p_{0}$, i.e.,

(4) $$\hat{m}\cdot t_{start}+\hat{q}=p_{0}$$

The obtained value $t_{start}$ represents the timestamp in the TS reference frame corresponding to the PPS event and is therefore used to synchronize the TS and GNSS datasets.

The uncertainty associated with the automatic synchronization procedure was estimated from the sensitivity of the fitted ramp-start time to the linear regression. In addition to the best-fit line, two limiting linear fits were considered, corresponding to the minimum and maximum slopes still compatible with the experimental ramp data. For each limiting line, the corresponding ramp-start time was computed as the intersection between the fitted line and the baseline position. The synchronization uncertainty was then estimated as the maximum deviation between these limiting start times and the start time obtained from the best-fit line. In the experimental configuration used in this work, this deviation was lower than 150 ms.

This uncertainty is mainly related to the limited number of TS samples available during the actuator motion and to the 5 Hz acquisition rate of the TS. A larger number of samples on the displacement ramp, for example obtained by reducing the actuator speed or increasing the actuator stroke, would allow the linear regression to be better constrained and could reduce the uncertainty of the automatic synchronization estimate.

**Figure 7:**
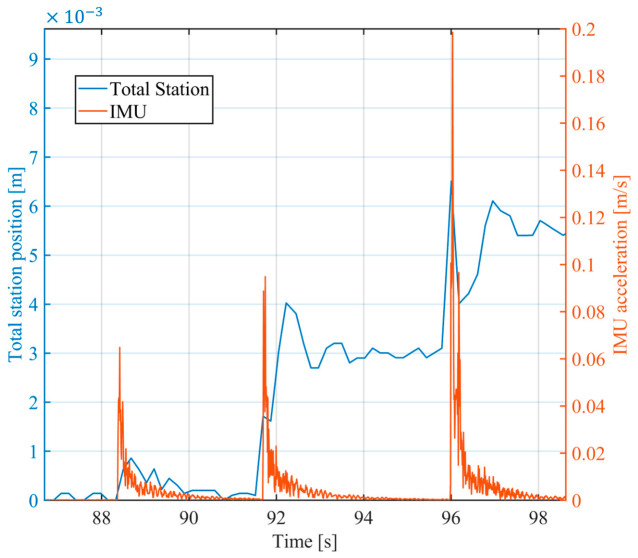
Comparison between the RP displacement measured by the TS and the acceleration recorded by the onboard IMU after applying the proposed synchronization procedure. The temporal correspondence between the displacement events and the acceleration peaks confirms the effectiveness of the PPS-based synchronization.

Figure 6 illustrates the synchronization procedure used to estimate $t_{start}$ during a laboratory experiment. The RP and the VESTA sensors were mounted on the drone, which was placed on a table. After the synchronization procedure was completed, the system was manually displaced three times. Figure 7 compares the displacement measured by the TS with the acceleration recorded by the onboard IMU after applying the synchronization method described above. A clear temporal correspondence between the abrupt displacement events and the acceleration peaks is evident, confirming the effectiveness of the proposed automatic synchronization procedure.

However, while the rising edges of the first event are perfectly aligned, a slight temporal offset can be observed for the second and third events, indicating that the synchronization process is affected by a certain degree of uncertainty. The uncertainty related to $t_{start}$ is smaller than the temporal resolution of the TS measurements (200 ms). Nevertheless, it may not be sufficient for an accurate comparison of high-frequency dynamic positioning data and could require an additional fine-tuning step.

### 2.3. Coordinate Transformation

The raw RTK, PPK, and TS measurements provide information on the drone trajectory; however, they refer to different physical points on the platform as shown in Figure 4. Specifically, the GNSS measurements correspond to the position of the onboard antenna, whereas the TS measurements refer to the position of the RP. To enable a meaningful quantitative comparison, these measurements must be referred to the same reference point. This requires accounting for the geometric offsets between the antenna and prism, as well as for the drone attitude variations occurring during flight. In this work, the drone was modeled as a rigid body. No dynamic model of the UAV was used for trajectory estimation, since the comparison is based on measured GNSS and TS positions. The mathematical model adopted in the processing is therefore a geometric lever-arm model, in which the position of the reflective prism is obtained from the GNSS antenna phase center by applying the attitude-dependent rotation of the known offset vector.

Since TS measurements are used as the reference dataset in this study, they were left unchanged, and the transformation was applied exclusively to the GNSS data. Accordingly, the RTK and PPK position estimates ($p_{GNSS}$) were projected from the antenna phase center to the prism reference point using the known offset distance vector $d_{i}$ and the attitude measurements provided by the VESTA system, as

(5) $$p_{prism}=p_{GNSS}+R\;d_{i},$$

where R is the rotation matrix defined by the attitude angles, and i=1,2 denotes the GNSS antenna. With reference to Figure 4, the offset vectors between GNSS antenna and RP were $d_{1}=\left(0,398,431\right)\;mm$ and $d_{2}=\left(0,-127,431\right)\;mm$ for GNSS1 and GNSS2 respectively. For this processing step, the RTK and PPK measurements, collected at 5 Hz, were interpolated onto the attitude time vector provided by the IMU, which was sampled at 100 Hz.

To correctly compute the rotation matrix in (5), the heading measured by the VESTA IMU must be aligned with the GNSS reference frame. After calibration, the IMU provides the yaw angle relative to the forward direction of the UAV body frame, rather than to a georeferenced horizontal frame. Therefore, an iterative procedure was developed to estimate the heading offset required to align the UAV heading with the North-East-Up (NEU) reference frame. The offset is obtained by minimizing the difference between the prism positions, $p_{prism}$, independently reconstructed from the two GNSS antennas.

The main physical and acquisition parameters of the experimental platform are summarized in Table 1. It is important to note that IMU platform acquires at 1 kHz. The data are filtered with an antialiasing filter at 500 Hz and processed with a Madgwick filter. After this processing, the data rate is 100 Hz.

## 3. Results

**Figure 8:**
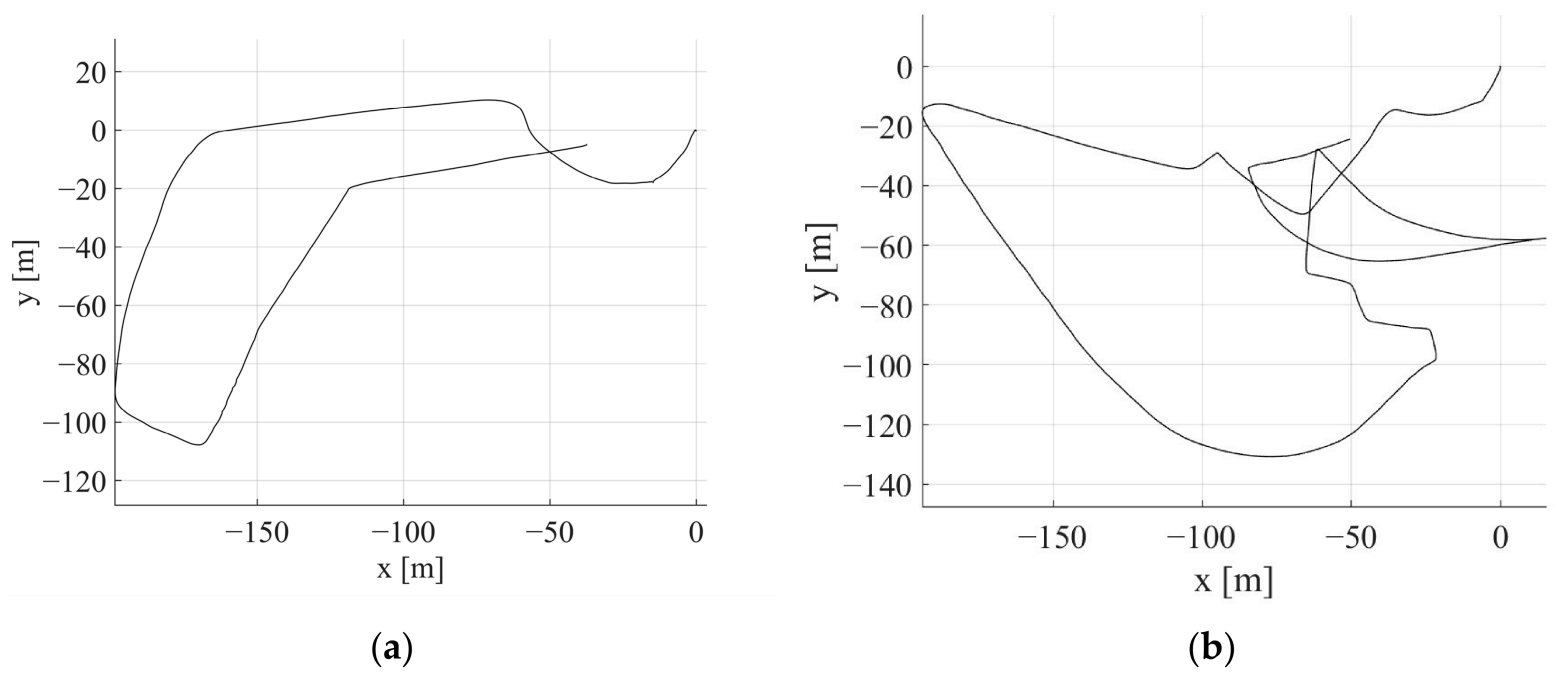
Drone trajectory reconstructed using TS measurements for two flights (**a**,**b**).

The flight tests were manually piloted in order to reproduce representative operating conditions for drone-based mapping and remote-sensing applications. The trajectories were not generated by an automatic trajectory planner; instead, they were intentionally selected to include different motion regimes, namely straight segments travelled at nearly constant speed, accelerated and decelerated segments, and curvilinear paths. The straight portions reproduce typical acquisition lines used in photogrammetric or radar-interferometric surveys, whereas the curved and accelerated portions were included to evaluate the positioning solutions under more demanding dynamic conditions. The PPS-synchronized TS trajectory of the RP was treated as the independent ground-truth reference. Figure 8a,b show the flight trajectories of two representative flights measured by the TS, visualized in the horizontal x-y plane.

**Figure 9:**
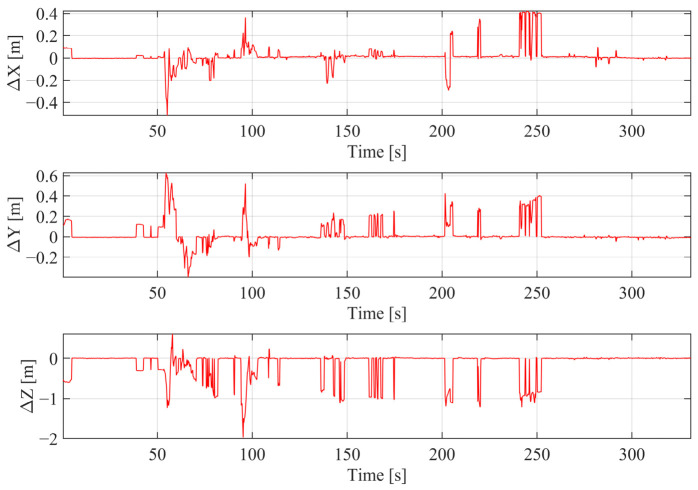
Difference between positioning coordinates measured using the PPK and RTK processing mode.

Figure 9 compares the two positioning solutions for flight (a), showing the differences between the PPK and RTK three orthogonal position components as a function of time. Significant discrepancies between the two solutions can be observed over several time intervals. The objective of this study was to determine which positioning method provides the most accurate estimate under dynamic operating conditions typical of drone applications. To this end, the GNSS position estimates were synchronized with the TS measurements and processed as described in the previous section, so as to refer the GNSS positions to the prism location and express both datasets in the same reference frame.

**Figure 10:**
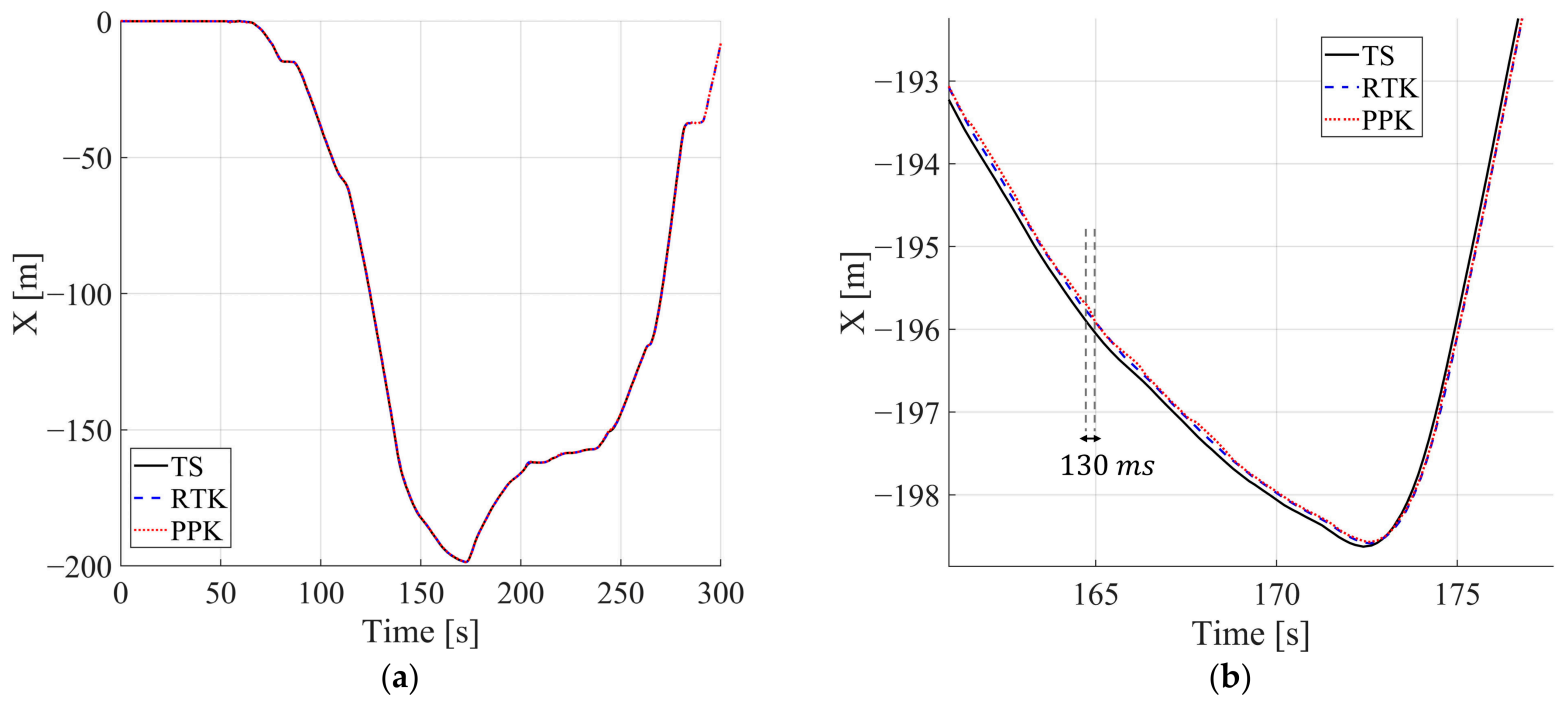
X position coordinate measured by TS, RTK and PPK solutions as a function of time of the whole flight (**a**), and of a specific section (**b**).

Following the automatic synchronization and the coordinate transformation to the prism position, the trajectories reconstructed from the TS, RTK, and PPK data show good agreement. Figure 10 presents the resulting x-coordinate for flight (a) as a function of time. Figure 10a shows the complete measurement, whereas Figure 10b reports an enlarged view of a representative time interval. After the automatic PPS-based synchronization, a small residual temporal offset was still visible between the TS trajectory and the RTK/PPK GNSS-derived trajectories. Several automatic refinement approaches, including cross-correlation and least-squares minimization of trajectory differences, were tested. However, these methods did not provide a stable estimate of the residual delay with an accuracy better than the 200 ms sampling interval of the TS and GNSS position measurements. This limitation is mainly due to the 5 Hz acquisition rate of both systems and to the fact that the RTK and PPK positioning errors, especially during degraded intervals, are not sufficiently correlated with the TS trajectory to allow an unambiguous automatic sub-sample alignment.

For this reason, the residual delay was estimated by visual inspection of one of the coordinates over a high-dynamic portion of the trajectory (in this case x-coordinate), where the sensitivity to timing errors is larger. The alignment was performed by simultaneously comparing the TS trajectory with both the RTK and PPK trajectories, and a single common delay was selected and applied to both positioning solutions. No independent delay optimization was performed for RTK and PPK. Therefore, this residual correction does not favor either positioning method, but only reduces the common timing offset remaining after the automatic PPS-based synchronization. The residual timing uncertainty is nevertheless considered a limitation of the experimental comparison. The residual delay used for flight (a) was 130 ms as shown in Figure 10b, for flight (b) the residual delay was 90 ms.

After the coordinate transformation (5), both RTK and PPK data are available at a sampling frequency of 100 Hz. Once the residual temporal offset has been removed, the TS measurements, originally acquired at 5 Hz, are interpolated onto the same time vector as the RTK and PPK data. This allows a point-by-point comparison between the GNSS and TS positions by computing the error vector

(6) $$\epsilon_{\begin{array}{c} \mathrm{RTK}\\ \mathrm{PPK} \end{array}}=p_{\begin{array}{c} \mathrm{RTK}\\ \mathrm{PPK} \end{array}}-p_{\mathrm{TS}},$$

where $p_{\begin{array}{c} \mathrm{RTK}\\ \mathrm{PPK} \end{array}}$ and $p_{\mathrm{TS}}$ are the three-dimensional position vectors provided by the GNSS solution and the TS, respectively. Considering the declared accuracy of the TS measurements, $\epsilon_{\begin{array}{c} \mathrm{RTK}\\ \mathrm{PPK} \end{array}}$ can be regarded as the positioning error affecting the RTK/PPK solutions.

**Figure 11:**
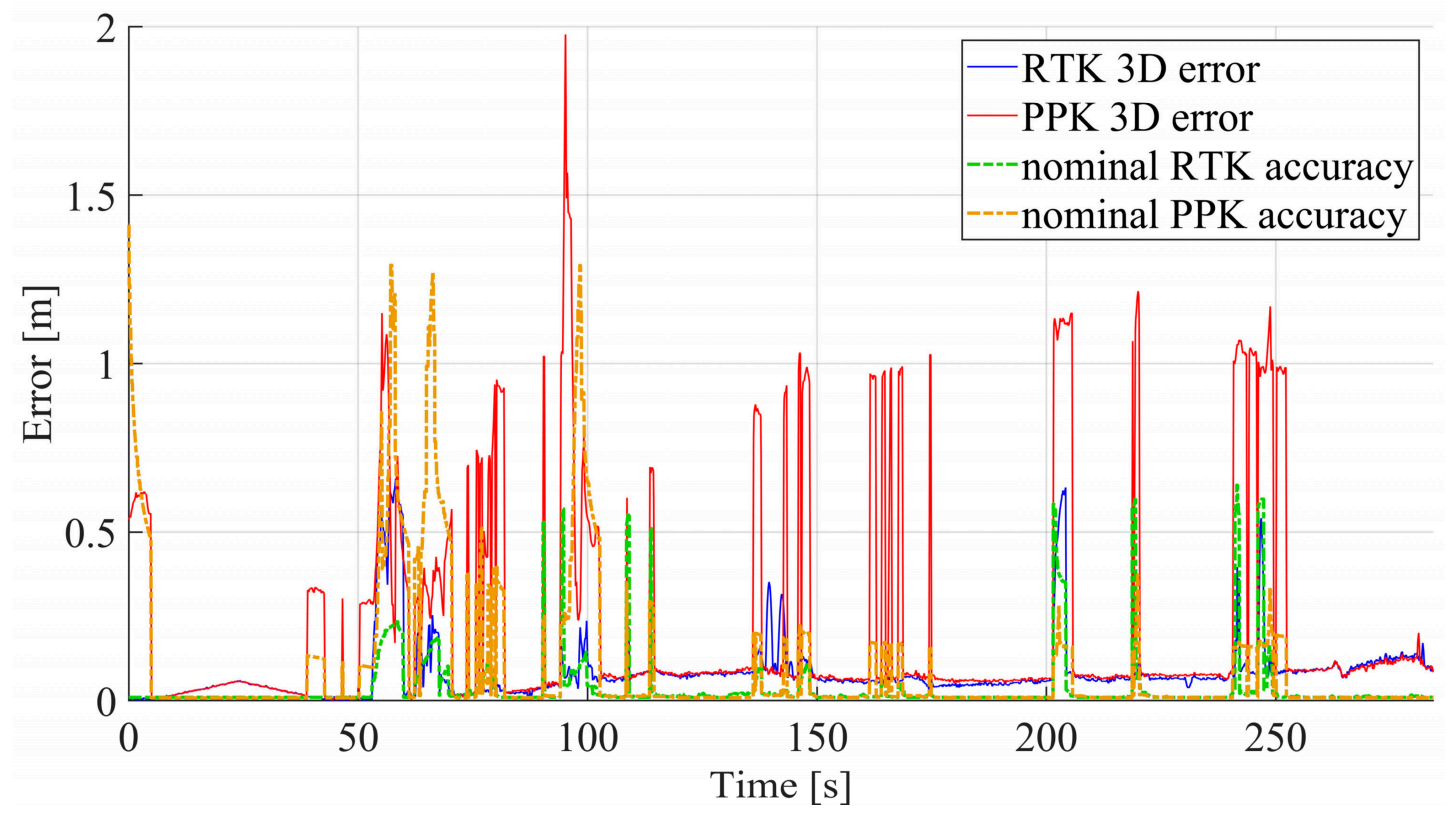
Magnitude of the positioning error vector for the RTK and PPK solutions during flight (a), compared with the corresponding positioning accuracy reported by the GNSS receiver.

Figure 11 compares the magnitude of the measured error vector for the RTK (blue) and PPK (red) solutions with the corresponding positioning accuracy declared by the receiver (green and yellow, respectively) for flight (a). The magnitude of the measured error, $\left|\epsilon_{\begin{array}{c} \mathrm{RTK}\\ \mathrm{PPK} \end{array}}\right|$, is defined as:

(7) $${\left|\epsilon_{\begin{array}{c} \mathrm{RTK}\\ \mathrm{PPK} \end{array}}\right|}^{2}=\epsilon_{x}^{2}+\epsilon_{y}^{2}+\epsilon_{z}^{2}.$$

Overall, the measured error follows the trend of the declared accuracy, although the correspondence is not exact. When the reported accuracy reaches its minimum value (0.01 m), both RTK and PPK exhibit measured errors of approximately 0.05 m. As the declared accuracy degrades, the measured error also increases. However, the measured error of the PPK solution often exceeds the corresponding declared accuracy. It is worth noting that, when the positioning quality is high, the RTK and PPK solutions provide comparable accuracy. Conversely, during degraded positioning conditions, the PPK solution generally exhibits larger errors than the RTK solution, occasionally exceeding 1 m. Furthermore, while the declared PPK accuracy appears to improve during the flight, the corresponding measured error remains approximately of the same magnitude.

**Figure 12:**
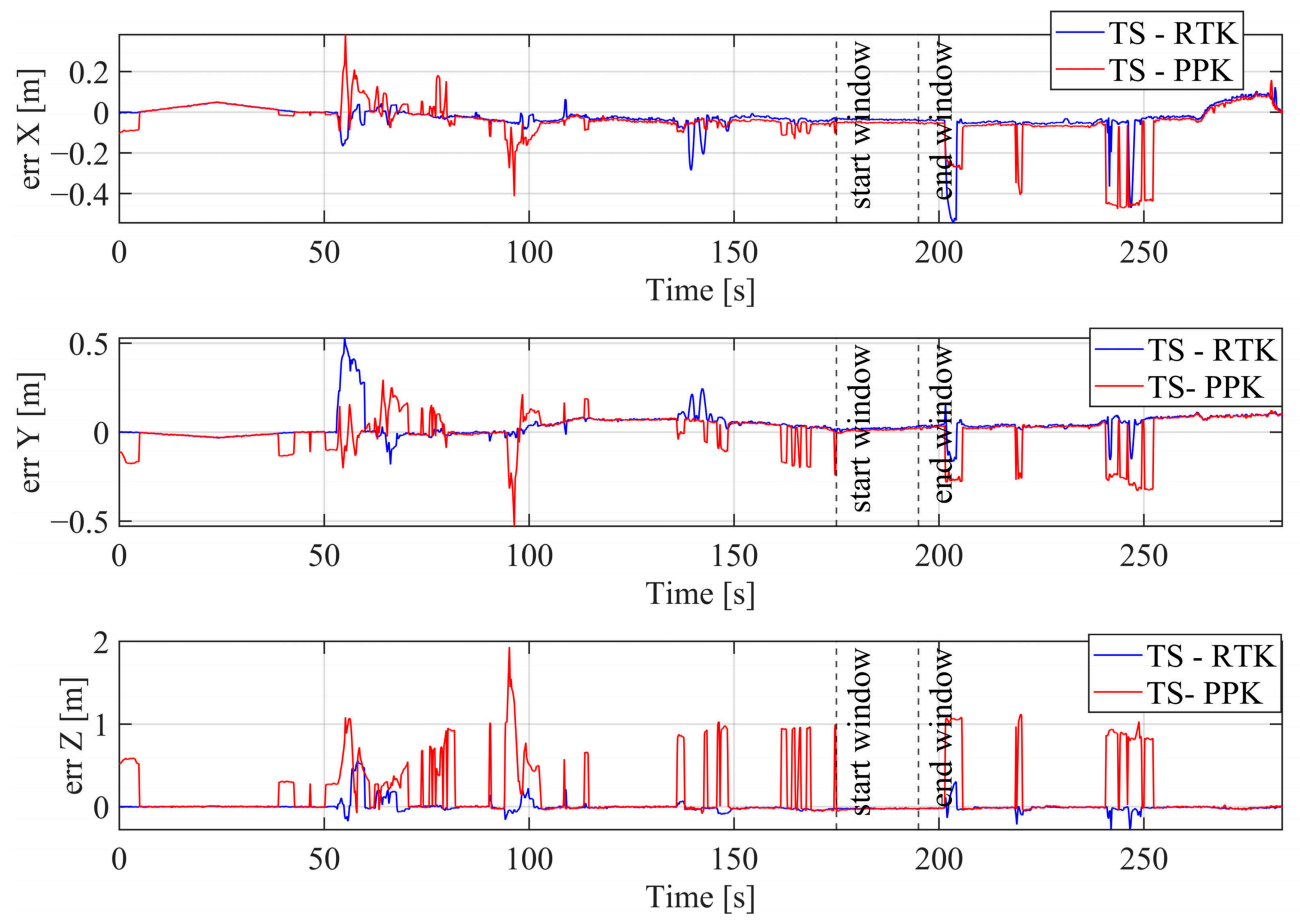
Positioning error components along the x, y, and z axes for the RTK and PPK solutions during the entire duration of flight (a). The dashed vertical lines indicate the time interval selected for the enlarged view shown in Figure 14.

**Figure 13:**
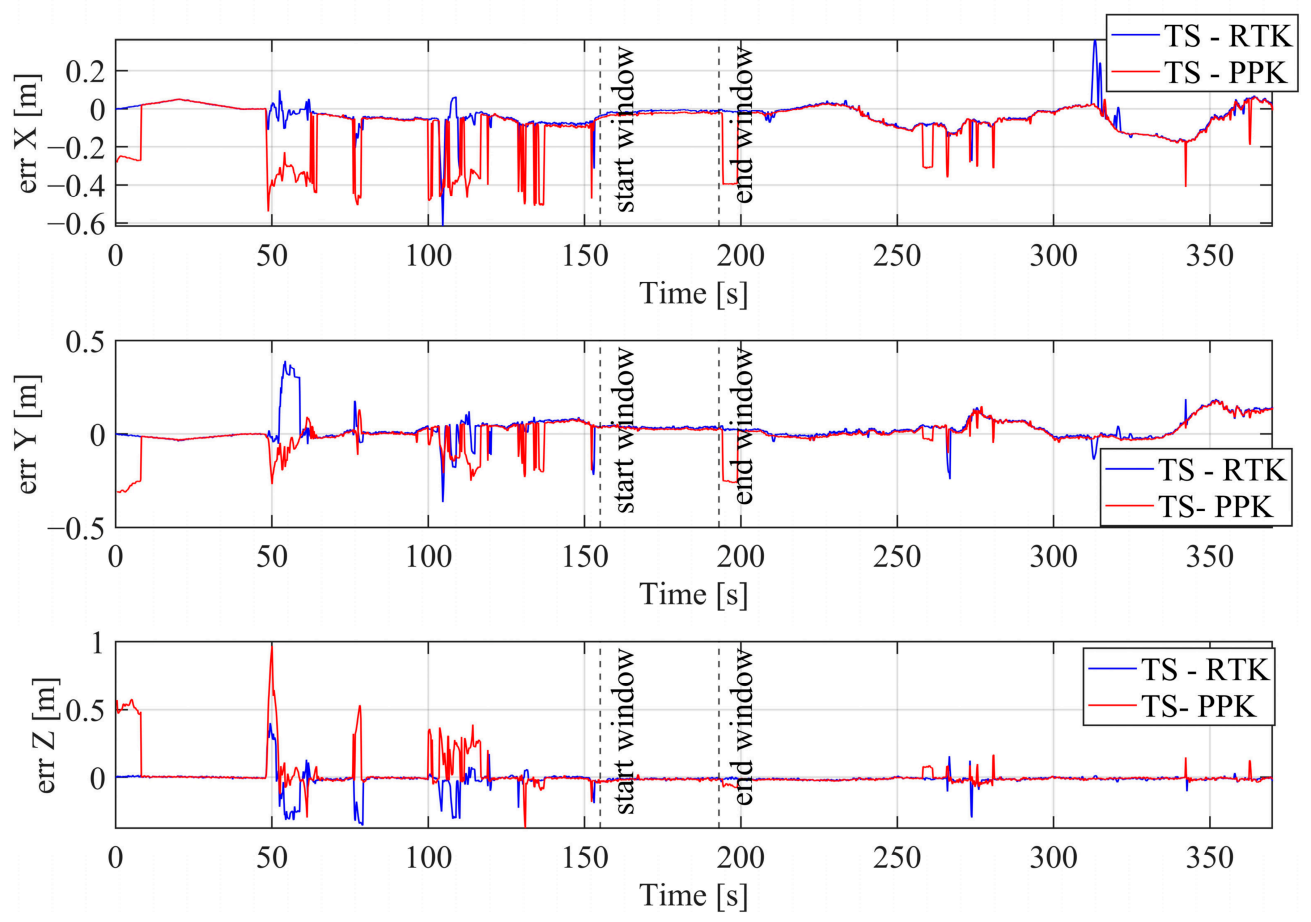
Positioning error components along the x, y, and z axes for the RTK and PPK solutions during the entire duration of flight (b). The dashed vertical lines indicate the time interval selected for the enlarged view shown in Figure 15.

Figure 12 and Figure 13 show the x-, y-, and z-components of the positioning error for the RTK (blue) and PPK (red) solutions obtained during flights (a) and (b), respectively. Several intervals can be identified in which the two methods provide nearly identical results; these correspond to periods in which the declared positioning accuracy reaches its best value. During degraded intervals, the reference RTKLIB-EX solution remains away from the reference trajectory for longer than the real-time u-blox solution, producing step-like PPK errors rather than the shorter RTK peaks.

**Figure 14:**
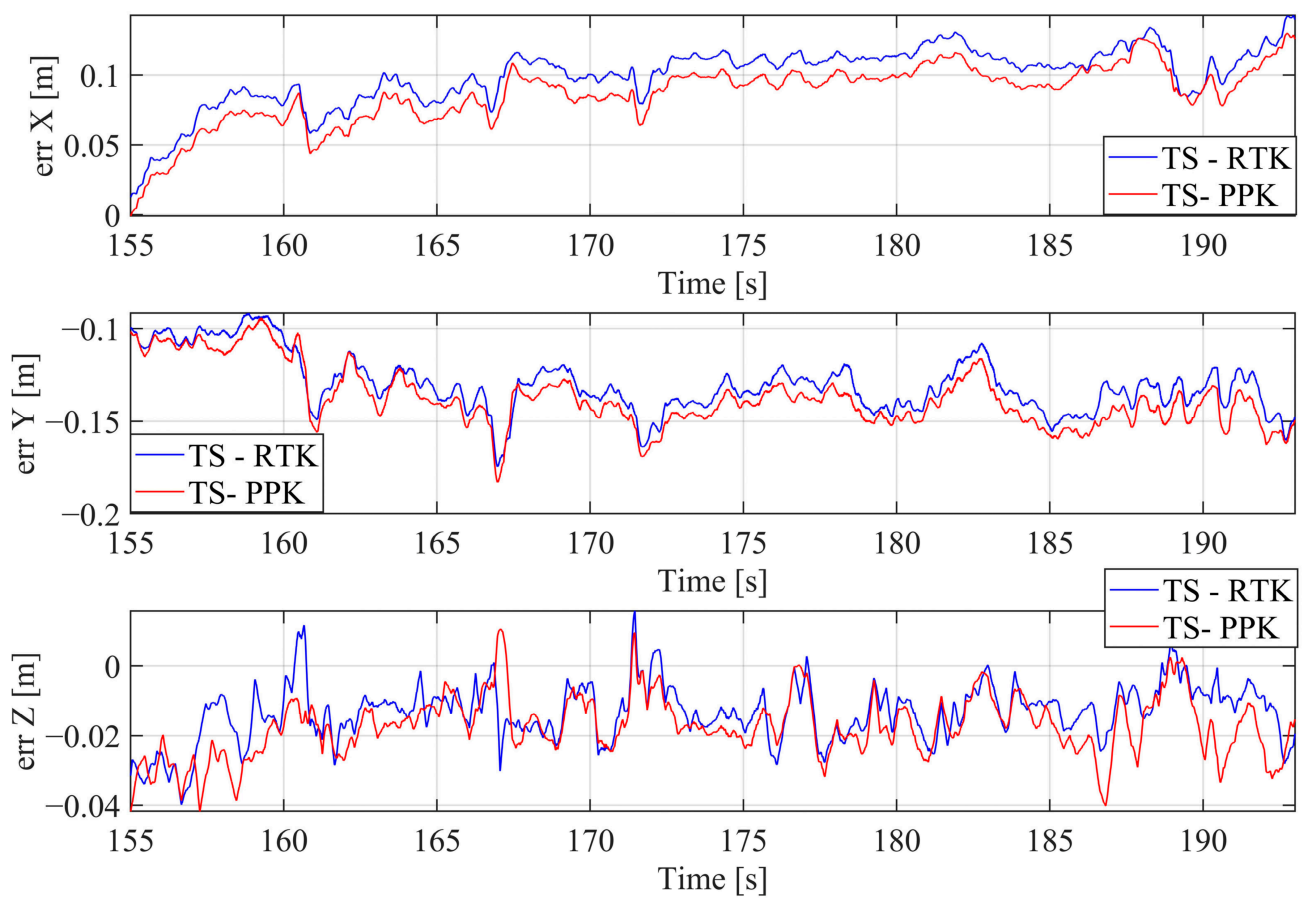
Enlarged view of the selected time interval highlighted in Figure 12. During this interval, both RTK and PPK solutions report their highest positioning accuracy, allowing a detailed comparison of the residual positioning errors.

**Figure 15:**
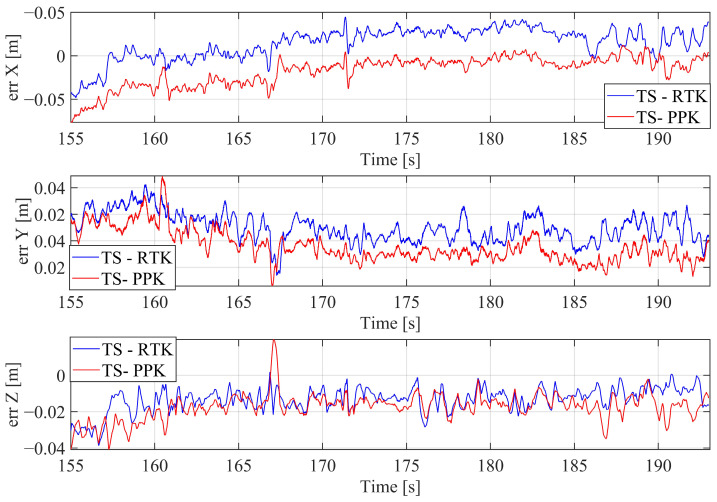
Enlarged view of the selected time interval highlighted in Figure 13. During this interval, the RTK and PPK solutions show comparable positioning performance under favorable GNSS conditions.

Figure 14 and Figure 15 report an enlarged view of the time interval highlighted by the dashed lines in Figure 12 and Figure 14, corresponding to periods in which both RTK and PPK report their highest positioning accuracy. During these intervals, the UAV follows an approximately straight trajectory at nearly constant velocity, representing particularly favorable operating conditions. Under these conditions, the two positioning methods exhibit comparable error magnitudes. Moreover, the three error components show similar temporal behavior for both RTK and PPK solutions, differing only by a small offset.

To quantitatively compare the performance of the two positioning methods, the root mean square error (RMSE) of the error vector was computed as

(8) $$\epsilon_{\begin{array}{c} \mathrm{RTK}\\ \mathrm{PPK} \end{array}}=\sqrt{mean\left({\left|\epsilon_{\begin{array}{c} \mathrm{RTK}\\ \mathrm{PPK} \end{array}}\right|}^{2}\right)},$$

where the error magnitude is defined in (7). In addition to the RMSE, the standard deviation (STD) of the error magnitude (7) was computed to quantify the dispersion of the positioning error around its mean value.

To address the behavior under degraded GNSS conditions, an additional statistical analysis was performed. High-accuracy intervals were defined as the samples for which the declared accuracy of all three position components was equal to or lower than 0.01 m. Conversely, degraded-accuracy intervals were defined as the samples for which at least one declared position accuracy component was higher than 0.01 m. This classification was based only on the receiver-reported accuracy and not on the TS error, avoiding the use of the ground-truth error in the interval selection.

Table 2 summarizes the RMSE values obtained for the RTK and PPK solutions under different evaluation conditions for both flights. The first column reports the RMSE calculated over the entire duration of each flight. In both datasets, the RTK solution outperforms the PPK solution, with RMSE values of 0.126 m and 0.118 m for flights (a) and (b), respectively, compared with 0.390 m and 0.205 m for the corresponding PPK solutions.

The second column reports that the RMSE computed over the high-accuracy in which the RMSE values of RTK and PPK solutions are very similar, and it is approximately 0.07 m. In terms of the degraded-accuracy interval, the difference between RTK and PPK is remarkable. The RME of RTK is lower than 0.15 cm, while for PPK it is 0.5 m and 0.25 for flight (a) and (b) respectively. Finally, the RMSE was also computed over the time windows shown in Figure 13 and Figure 15, where both positioning methods exhibit errors of approximately 0.05 m. These intervals were selected because they are representative of waypoint-to-waypoint flight segments, in which the UAV moves with approximately constant speed and direction.

Table 3 reports the STDs of the error magnitude computed over the different evaluation intervals. The STD quantifies the dispersion of the error magnitude defined in (7) within each interval. When the entire flight and the degraded-accuracy intervals are considered, the STD associated with the PPK solution is approximately one order of magnitude larger than that obtained for the RTK solution. In contrast, during high-accuracy intervals and selected time windows, the STD values of the two positioning solutions are comparable.

Reprocessing the same raw rover and base observations showed that the RTKLIB-EX settings can affect PPK performance substantially. Reducing the elevation mask from 15° to 10° reduced the entire-flight RMSE by 0.037 m in flight (a) and by 0.027 m in flight (b). The 20° mask increased the RMSE of flight (a) by 0.231 m, while fix-and-hold reduced the RMSE of flight (b) by 0.034 m but provided no improvement in flight (a). Restricting the processing to GPS alone caused the largest deterioration and markedly reduced the percentage of fixed epochs. In the controlled GPS-only comparison, replacing broadcast ephemerides with IGS final precise ephemerides changed the entire flight RMSE by only 0.09 mm in flight (a) and 0.25 mm in flight (b), with essentially unchanged fixed-solution percentages. Thus, precise ephemerides had no material effect for these short-baseline datasets. Of the tested variants, only the 10° continuous multi-GNSS configuration consistently improved both flights without reducing the fixed-solution percentage; however, its entire-flight RMSE values (0.360 m and 0.178 m) remained higher than those of RTK (0.126 m and 0.118 m) in flights (a) and (b), respectively. Table 4 summarizes the comparison between the different RTKLIB-EX settings. The ΔRMSE in Table 4 represents the differences between the RMSE of reference setting (Broadcast, multi-GNSS, 15°, continuous) and the other settings.

Table 4 reports the percentage of epochs associated with the best quality flag (Q = 1) for each selected processing configuration. It is worth noting that, when a multi-GNSS configuration is used, the percentage of fixed solutions is higher than 79%, whereas in the GPS-only configuration it drops below 40%.

However, the quality flag does not always directly reflect the actual positioning accuracy. For example, in the reference configuration, 5.35% of the epochs in flight (a) and 1.18% of the epochs in flight (b) were classified with the best quality flag (Q = 1), but exhibited an RMSE larger than 0.25 m. These intervals were very short: the longest periods in which the PPK solution simultaneously showed the best quality flag and a degraded positioning error (Q = 1 and RMSE ≥ 0.25 m) lasted 0.98 s in flight (a) and 0.04 s in flight (b). All other intervals with degraded positioning error were associated with a poorer quality flag (Q > 1). Therefore, the quality flag may occasionally produce false positives, i.e., epochs classified as fixed despite a degraded positioning error. However, in the analyzed dataset, Q > 1 consistently indicated a low-precision solution, and no persistent incorrect-fix events were observed.

The persistence of the best quality flag depends on the RTKLIB-EX processing settings. In the present analysis, the best-performing configuration in terms of fixed solutions with error magnitude lower than 0.25 m was “Broadcast, multi-GNSS, 10°, continuous”, whereas the worst-performing configuration was “Broadcast, multi-GNSS, 15°, fix-and-hold”. The observed recovery behavior is therefore specific to the adopted processing configuration and dataset, rather than an intrinsic limitation of PPK.

## 4. Discussion

The aim of this study was to compare the performance of RTK and PPK positioning solutions, which offer different advantages depending on the intended application. PPK does not require communication infrastructures, such as dedicated radio links, and is therefore particularly suitable for operations in areas with limited or unreliable network connectivity. In addition, because the GNSS observations are processed offline, the positioning solution can be recomputed multiple times using different processing parameters or algorithms, providing greater flexibility during data analysis. In contrast, RTK provides real-time positioning, enabling immediate assessment of the positioning quality during the survey. This allows the operator to verify whether the GNSS solution has converged to its highest accuracy and to identify potential positioning issues as they occur. Conversely, when operating in PPK mode, the quality of the positioning solution can only be assessed after the survey has been completed and the data has been post-processed.

The present experiment was carried out in a short-baseline configuration, in which both RTK corrections and PPK processing relied on the same local base station. This configuration was intentionally adopted to provide a controlled comparison between the two positioning strategies under the same GNSS conditions. Although RTK and PPK can both provide high-accuracy solutions over longer baselines when the correction link and GNSS conditions are adequate, the present work does not include a direct TS validation for such scenarios.

Accurate synchronization between the GNSS and TS measurements is a key requirement for a reliable comparison of the two positioning methods. The synchronization strategy proposed in this work provides automatic temporal alignment with an estimated uncertainty of approximately less than 150 ms. This value is smaller than the temporal resolution of the TS measurements (200 ms, corresponding to the 5 Hz acquisition frequency), demonstrating that the proposed approach is sufficiently accurate for the synchronization task. Nevertheless, for the purpose of evaluating positioning errors at the centimetric level, this residual uncertainty still contributes to the estimated error. For this reason, an additional fine-tuning procedure was applied to further improve the temporal alignment between the two datasets. It should be noted that the residual delay (130 ms for flight (a) and 90 ms for flight (b)) was estimated from the comparison between the TS and GNSS-derived trajectories and not from an additional independent timing reference. Therefore, this correction should not be interpreted as a further improvement of the intrinsic accuracy of the automatic PPS-based synchronization procedure. Instead, it was used as a common post-processing adjustment to reduce the residual temporal offset before computing the positioning errors. The same correction was applied to both RTK and PPK solutions, and no method-dependent time shift was introduced. For this reason, the correction is not expected to affect the relative comparison between RTK and PPK. Nevertheless, the residual uncertainty of the temporal alignment remains a limitation of the experimental setup and may contribute to the absolute positioning error estimated with respect to the TS reference.

The synchronization accuracy could be further improved by increasing the number of samples used in the linear regression employed to estimate the synchronization instant. Since the actuator length is constrained by physical limitations, this could be achieved by reducing the actuator velocity, thereby increasing the number of samples acquired during the synchronization procedure. However, even under these conditions, noise measurement would still introduce a residual uncertainty in the estimated synchronization time. Consequently, additional refinement techniques are expected to remain necessary for applications requiring high-precision performance evaluation. Finally, although the TS trajectory was interpolated onto the 100 Hz time vector used for the comparison, both TS and GNSS position measurements were originally acquired at 5 Hz. Therefore, the interpolation only provides a common time vector for the error computation and does not increase the temporal information content of the measurements. As a consequence, the proposed validation should be interpreted as representative of trajectory errors and transient deviations occurring at time scales compatible with the 5 Hz positioning data.

For the tested short-baseline datasets, the real-time u-blox solution achieved lower entire-flight RMSE than the reference RTKLIB-EX PPK solution, although the two methods remained comparable during optimal reported-accuracy intervals. The ambiguity diagnostics show that the longer recovery observed for the reference RTKLIB-EX solution was mainly associated with prolonged float or degraded differential states, consistent with the time required to re-establish and validate the carrier-phase ambiguities; it did not generally indicate a stable but incorrectly fixed integer solution. Moreover, among the tested variants, the 10° continuous multi-GNSS configuration was the only setting that improved the entire-flight RMSE in both flights without decreasing the fixed-solution percentage, whereas other parameter changes could improve one flight while degrading another. Although it was the most robust PPK setting tested, it did not outperform RTK over the entire flights. This identifies the most robust configuration within the tested set, rather than a universally optimal elevation mask. Accordingly, these results characterize the specific receiver, RTKLIB-EX configuration, and datasets considered here and should not be interpreted as an intrinsic performance ranking of all RTK and PPK implementations.

Future work will focus on extending the experimental validation to longer baselines and to third-party reference stations, in order to evaluate the influence of base-station distance and correction infrastructure on RTK and PPK performance. Further work will extend the present sensitivity analysis to additional ambiguity-validation thresholds, cycle-slip handling strategies, and multi-GNSS precise products. From the synchronization perspective, the uncertainty of the TS alignment could be reduced by increasing the number of samples acquired during the actuator motion or by adopting higher-rate external tracking systems. Finally, the proposed PPS-based synchronization framework is not limited to multicopter UAVs and could be extended to other mobile robotic platforms, such as fixed-wing UAVs, VTOL platforms, ground vehicles, or marine vehicles, as well as to additional scientific payloads requiring accurate time alignment.

Table 5 summarizes the main characteristics, advantages, and limitations of RTK and PPK positioning for drone applications.

## 5. Conclusions

This work presented a methodology for the direct evaluation of UAV positioning accuracy based on a PPS-synchronized robotic TS used as an independent reference system. The proposed synchronization and processing strategy enables a point-by-point comparison between GNSS-derived positions and ground-truth measurements under dynamic flight conditions, providing a reliable framework for assessing the performance of different positioning techniques.

The synchronization procedure developed in this study allowed the TS measurements to be aligned with the GNSS time reference with a residual uncertainty lower than 150 ms. Although this value is smaller than the temporal resolution of the TS measurements, it still represents a non-negligible contribution when centimeter-level positioning errors are evaluated. For this reason, a fine-tuning procedure was applied to further reduce the residual temporal offset before the comparison between GNSS and Total Station data.

The experimental results showed that RTK and PPK provide comparable accuracy when the reported GNSS positioning quality is optimal. In these conditions, both solutions exhibited positioning errors of approximately 0.05 m with respect to the TS reference. However, when the entire flight trajectories were considered, the RTK solution achieved lower RMSE values than the PPK solution. In particular, RMSE values of 0.126 m and 0.118 m were obtained for RTK in the two analyzed flights, whereas the corresponding PPK values were 0.390 m and 0.205 m.

For the short-baseline experiments and the specific u-blox and RTKLIB-EX implementations considered in this study, the real-time solution achieved lower entire-flight RMSE than the post-processed solution. Following GNSS degradation, the PPK solution remained in float or degraded states for longer before a validated fixed solution was recovered, producing larger transient errors than the RTK solution. The sensitivity analysis on PPK settings further showed that precise ephemerides produced a negligible change in the controlled GPS-only comparison, whereas the elevation mask, constellation selection, and ambiguity-resolution strategy could materially affect the result. The findings therefore do not imply that RTK inherently outperforms PPK, and the processing configuration and operating conditions must be considered when selecting and interpreting either solution.

## Figures and Tables

**Table 1:** Main physical and acquisition parameters of the experimental platform.

Parameter	Value
GNSS antenna baseline	525 mm
Linear actuator stroke	60 mm
GNSS1—RP offset	$\left(0,398,431\right)\;mm$
GNSS2—RP offset	$\left(0,-127,431\right)\;mm$
TS sampling frequency	5 Hz
GNSS positioning rate	5 Hz
IMU sampling frequency	1 kHz
IMU data rate	100 Hz

**Table 2:** Comparison between RMSE of RTK and PPK solutions obtained under different evaluation conditions for flight (a) and (b).

		RMSE [m]			
Processing Mode	Flight	Entire Flight	High Accuracy	Degraded Accuracy	Selected Window
RTK	(a)	0.126	0.072	0.147	0.048
	(b)	0.118	0.081	0.135	0.042
PPK	(a)	0.390	0.077	0.479	0.061
	(b)	0.205	0.073	0.250	0.044

**Table 3:** Comparison between STD of (7) of RTK and PPK solutions obtained under different evaluation conditions for flight (a) and (b).

		STD [m]			
Processing Mode	Flight	Entire Flight	High Accuracy	Degraded Accuracy	Selected Window
RTK	(a)	0.095	0.03	0.114	0.004
	(b)	0.082	0.051	0.093	0.008
PPK	(a)	0.319	0.03	0.370	0.004
	(b)	0.158	0.041	0.184	0.009

**Table 4:** Sensitivity of the PPK solution to RTKLIB-EX processing settings. ΔRMSE represents the differences between the RMSE of reference setting (Broadcast, multi-GNSS, 15°, continuous) and the other settings. Fixed denotes the percentage of RTKLIB solution epochs classified as Q = 1.

	Flight (a)	Flight (a)	Flight (b)	Flight (b)
PPK Configuration	ΔRMSE [m]	Fixed [%]	ΔRMSE [m]	Fixed [%]
Broadcast, multi-GNSS, 15°, continuous (reference configuration)	0.000	79.6	0.000	86.3
Broadcast, multi-GNSS, 10°, continuous	−0.037	79.7	−0.027	89.1
Broadcast, multi-GNSS, 20°, continuous	+0.231	80.7	0.000	86.3
Broadcast, multi-GNSS, 15°, fix-and-hold	+0.003	88.2	−0.034	89.8
Broadcast, GPS-only, 15°, continuous	+0.246	37.9	+0.329	9.9
IGS final precise, GPS-only, 15°, continuous	+0.246	37.9	+0.329	9.8

**Table 5:** Summary of the main characteristics, advantages, and limitations of RTK and PPK positioning for drone applications.

Aspect	RTK	PPK
Processing strategy	Real-time differential positioning	Offline post-processing of raw GNSS observations
Communication requirement	Requires real-time correction link	No real-time correction link required during flight
Position availability	Available during the mission	Available only after post-processing
Operational advantage	Allows immediate monitoring of positioning quality	Allows reprocessing with different parameters or base stations
Main limitation	Depends on the reliability of the correction link	Positioning quality can be assessed only after the flight
Observed behavior in this study	Lower RMSE over the complete dynamic trajectories	Comparable accuracy in optimal conditions; with reference RTKLIB-EX configuration, longer float/degraded intervals after GNSS degradation

## Data Availability

The raw data supporting the conclusions of this article will be made available by the authors on request.

## Abbreviations

GCP	Ground Control Point
GNSS	Global Navigation Satellite System
IMU	Inertial Measurement Unit
NEU	North-East-Up
PPK	Post-Processed Kinematic
PPS	Pulse-Per-Second
RF	Radio Frequency
RINEX	Receiver INdependent EXchange format
RMSE	Root Mean Square Error
ROS	Robot Operating System
RP	Reflective Prism
RTK	Real-Time Kinematic
RTKLIB	Open-source GNSS positioning software package
STD	Standard deviation
TS	Total Station
UART	Universal Asynchronous Receiver-Transmitter
UAV	Unmanned Aerial Vehicle
UTC	Coordinated Universal Time
VESTA	Versatile External Synchronization and Telemetry Architecture
